# Insight into broad substrate specificity and synergistic contribution of a fungal α-glucosidase in Chinese Nong-flavor daqu

**DOI:** 10.1186/s12934-023-02124-z

**Published:** 2023-06-15

**Authors:** Zhuolin Yi, Lanchai Chen, Yanling Jin, Yi Shen, Nian Liu, Yang Fang, Yao Xiao, Xi Wang, Kui Peng, Kaize He, Hai Zhao

**Affiliations:** 1 CAS Key Laboratory of Environmental and Applied Microbiology, Environmental Microbiology Key Laboratory of Sichuan Province, Chengdu Institute of Biology, Chinese Academy of Sciences, No. 9 Section 4, Renmin Nan Road, Chengdu, Sichuan 610041 P.R. China; 2 School of Food and Bioengineering, Xihua University, Chengdu, Sichuan 610039 China; 3 Sichuan Langjiu Co., Ltd, Gulin, 646523 China; 4 Sichuan Food and Fermentation Industry Research & Design Institute, Chengdu, 611130 China; 5 Analytical and Testing Center, Sichuan University of Science and Engineering, Zigong, 643000 China

**Keywords:** α-Glucosidase (AG), Liquor starter, Heterologous expression, Glycoside hydrolase family 31 (GH31), Metatranscriptomics

## Abstract

**Background:**

Chinese Nong-favor daqu, the presentative liquor starter of Baijiu, has been enriched with huge amounts of enzymes in degrading various biological macromolecules by openly man-made process for thousand years. According to previous metatranscriptomics analysis, plenty of α-glucosidases were identified to be active in NF daqu and played the key role in degrading starch under solid-state fermentation. However, none of α-glucosidases was characterized from NF daqu, and their actual functions in NF daqu were still unknown.

**Results:**

An α-glucosidase (NFAg31A, GH31-1 subfamily), the second highest expressed α-glucosidases in starch degradation of NF daqu, was directly obtained by heterologous expression in *Escherichia coli* BL21 (DE3). NFAg31A exhibited the highest sequence identities of 65.8% with α-glucosidase II from *Chaetomium thermophilum*, indicating its origin of fungal species, and it showed some similar features with homologous α-glucosidase IIs, i.e., optimal activity at pH ~ 7.0 and litter higher temperature of 45 ℃, well stability at 41.3 ℃ and a broad pH range of pH 6.0 to pH 10.0, and preference on hydrolyzing Glc-α1,3-Glc. Besides this preference, NFAg31A showed comparable activities on Glc-α1,2-Glc and Glc-α1,4-Glc, and low activity on Glc-α1,6-Glc, indicating its broad specificities on α-glycosidic substrates. Additionally, its activity was not stimulated by any of those detected metal ions and chemicals, and could be largely inhibited by glucose under solid-state fermentation. Most importantly, it exhibited competent and synergistic effects with two characterized α-amylases of NF daqu on hydrolyzing starch, i.e., all of them could efficiently degrade starch and malto-saccharides, two α-amylases showed advantage in degrading starch and long-chain malto-saccharides, and NFAg31A played the competent role with α-amylases in degrading short-chain malto-saccharides and the irreplaceable contribution in hydrolyzing maltose into glucose, thus alleviating the product inhibitions of α-amylases.

**Conclusions:**

This study provides not only a suitable α-glucosidase in strengthening the quality of daqu, but also an efficient way to reveal roles of the complicated enzyme system in traditional solid-state fermentation. This study would further stimulate more enzyme mining from NF daqu, and promote their actual applications in solid-state fermentation of NF liquor brewing, as well as in other solid-state fermentation of starchy industry in the future.

**Supplementary Information:**

The online version contains supplementary material available at 10.1186/s12934-023-02124-z.

## Background

Baijiu (Chinese liquor), one of the oldest distilled spirits in the world, has been produced for thousands of years, and Nong-favor (NF, also called strong aroma type) baijiu accounts for about 70% of baijiu production [1]. NF daqu, liquor starter of NF baijiu, is openly and manually made by solid-state fermentation, with wheat, barley and/or peas as its material [2, 3]. Thus, special and various microbial community are continually and stably enriched in NF daqu [4, 5], by which huge amounts of enzymes would be secreted to efficiently degrade components of material, i.e., starch, protein, glucan, cellulose and hemicellulose [6–9].

Starch is the highest ingredients of cereal material in NF daqu, and its degradation requires synergistic actions among various enzymes, such as α-amylase, β-amylase, glucoamylase, α-glucosidase, isoamylase and pullulanase [10], making the largest contribution to the liquefaction and saccharification capacities of NF daqu [6]. Among them, α-amylases, glucoamylases, and α-glucosidases are the abundant enzymes involved starch degradation in NF daqu, and α-glucosidases are identified as the most abundant enzymes in starch degradation by metagenomic study [6]. Moreover, based on metatranscriptomics, the largest number of 932 carbohydrate-active enzymes was detected at the high temperature stage (N3) of NF daqu, and plenty of α-glucosidases, together with α-amylases and glucoamylases, are highly active at N3 [9]. According to our unpublished data, a total of 14 α-glucosidases were identified at N3 of NF daqu, which belong to glycoside hydrolase (GH) family GH31. To date, only two α-amylases (NFAmy13A and NFAmy13B) were directly characterized from NF daqu in our group [11, 12], and no more study is reported on the characterization of α-glucosidases from NF daqu, or other liquor starter. Thus, details about the function of α-glucosidases in the complicated enzyme system of NF daqu was still unknown.

α-Glucosidase (AG) (EC 3.2.1.20) could hydrolyze α-1,4-glucosidic linkages to release glucose from non-reducing end via an exo-acting manner [13–15]. AGs are widely distributed in most organisms, such as animals, plants, fungi, bacteria, and archaea, indicating their pivotal roles in physiological starch and glycogen metabolisms, which produce glucose in the amylolytic pathway. Based on substrate specificity, this enzyme can be classified into three main groups, including type I, II, and III [16]. Type I AGs prefer the degradation of heterogeneous linkages (e.g., sucrose and *p*-nitrophenyl-α-D-glucopyranoside (pNPG)), whereas Type II and III AGs prefer the hydrolysis of homogeneous linkages (e.g., maltose, maltooligosaccharides, and starch). Between Type II and III AGs, Type III AGs show higher efficiency on degrading polysaccharides, such as starch. According to the CAZy classification, AG (EC 3.2.1.20) are reported in GH4, GH13, GH31, GH63, GH97, and GH122, and mainly belong to GH13 and GH31. AG in GH31 family prefers the degradation of homogeneous substrates (type II and Type III), whereas AG in GH13 family prefers the degradation of heterogeneous substrates (type I) [16]. Most importantly, AG in GH31 family primarily hydrolyzes α-1,4 glucoside bonds and could function efficiently with glucoamylase and α-amylase on starch degradation [17]. Thus, taking into account that 14 α-glucosidases from N3 of NF daqu all belong to GH31 family, α-glucosidases in GH31 should definitely play the key role in starch degradation of NF daqu. In addition, besides the major function of degrading α-1,4 glycosidic bonds, the characterized GH31 family members also harbor other hydrolytic activities, such as α-glucosidase II [14, 18], α-1,3-glucosidase [13], α-xylosidase [19], α-galactosidase [20], maltase-glucoamylase (MGAM) [21], sucrase-isomaltase (SI) [22], sulfoquinovosidase (Sul) [23], α-N-acetylgalactosaminidases (AaGal) [24, 25].

NF daqu is a typic medium-temperature daqu, which must be maintained at a relatively high temperature of 50 − 60 °C for more than one week during its making process [1, 9]. According to previous metatranscriptomics analysis, starch-degrading enzymes of α-glucosidases, α-amylases and glucoamylases were all highly active at this high temperature stage (N3) of NF daqu, and α-glucosidases, containing 14 members, were the first highest expressed ones, with RPKM (Reads Per Kilobase per Million) value of 937.3 at N3 [9], which could definitely indicate the pivotal function of α-glucosidases in NF daqu. Among 14 α-glucosidase genes, several genes showed relatively high expression levels with RPKM values from 14.8 to 655.5, especially two gene products, which stand for the 1th and 25th highest expression of carbohydrate-active enzymes at N3 [9]. Therefore, α-glucosidases with high expression levels are selected for further characterization. Finally, one fungal α-glucosidase, the second highest expressed α-glucosidases with RPKM value of 127.1, was similarly obtained from NF daqu by a metatranscriptomics-based method, and a thorough analysis was performed on its enzymatic properties, as well as its possible synergistic effects with two previously characterized α-amylases (NFAmy13A and NFAmy13B), so as to get close to their actual functions in solid-state system of NF daqu, and promote their applications in strengthening quality of daqu in the future.

## Methods

### Materials

*Escherichia coli* Trans5ɑ and BL21(DE3) (TransGen Biotech, Beijing, China) was used for plasmid maintenance and protein expression, respectively. T4 DNA ligase, *Nde*I, *Hind*III (New England Biolabs, Ipswich, MA, USA) and pET-28a + vector (Invitrogen, Carlsbad, CA, USA) were used for gene cloning. Ni-NTA Sefinose™ Resin was purchased from Sangon Biotech (Shanghai, China) for protein purification. *p*-Nitrophenyl-α-D-glucopyranoside (*p*NPαG), *p*-Nitrophenyl-β-D-glucopyranoside (*p*NPβG), *p*-Nitrophenyl-β-D-xylopyranoside (*p*NPβX), *p*-Nitrophenyl-α-D-mannopyranoside (*p*NPαM), and *p*-Nitrophenyl-α-L-arobinopyranoside (*p*NPαA) were from *J&K* Scientific (Beijing, China), while soluble starch, malto-oligosaccharides, Nigerose, Kojibiose, Isomaltose, Sucrose, Kanamycin sulphate, imidazole, isopropylthio-β-galactoside (IPTG) and other reagents were purchased from yuanye Bio-Technology (Shanghai, China).

### Gene cloning, expression and protein purification

Since the gene product of ORF15963 was the first characterized alpha-glucosidase in the GH31 family from NF daqu, it was designated as NFAg31A. The gene fragment of NFAg31A, excluding the signal peptide, was directly amplified from cDNA library of NF daqu by Mix (Green) (TsingKe, Beijing, China), with two specific primers of NFAg31AF 5’CATATGGTGAAGCATGAAAACTTCAAGAAATG 3’ and NFAg31AR 5’AAGCTTCTAGATCCTCCACTCCCTG3’ containing *Nde*I and *Hind*III site, respectively. The PCR products of *NFAg31A* and pET-28a + vector were both cleaved by *Nde*I and *Hind*III, and their purified DNA fragments were ligated with T4 DNA ligase (New England Biolabs). The recombinant plasmid of pET-28a+-*NFAg31A* was then transformed into *E.coli* Trans5ɑ, and transformants were sprayed onto Luria-Bertani (LB) medium with kanamycin (50 µg/mL). Single colonies were re-cultured into LB medium with same antibiotics overnight, and plasmid was extracted from culture with Qiagen mini-prep kit accordingly. Finally, the recombinant plasmid with the correct inserts was verified by DNA sequencing (Sangon Biotech).

Next, the recombinant protein of NFAg31A was produced in *E.coli* BL21(DE3). In short, 1 mL pre-culture of *NFAg31A* transformants was inoculated into 1 L of LB medium with same antibiotics, and cultured at 37 ℃. When the culture reached an optical density of 0.6 at 600 nm, the expression was induced with 0.05 mM IPTG at 25 ℃ for 48 h. The cells were collected by centrifugation at 4 ℃, suspended in binding buffer (50 mM Tris-HCl, 300 mM NaCl, pH 7.5), and disrupted by sonication with Sonics Vibra-Cell™ (Sonics & Materials, Newtown, USA). The ruptured cells were clarified by centrifugation at 4 ℃, and the resulting supernatant was then applied to protein purification.

NFAg31A with C-terminal 6xHis-tag was adsorbed on Ni-NTA Sefinose™ resin, washed by binding buffer containing 20 mM imidazole, and eluted by binding buffer containing 200 mM imidazole. The elution was successively collected in fraction, and analyzed by sodium dodecyl sulfate-polyacrylamide gel electrophoresis (SDS-PAGE). Fractions with the purified proteins were finally concentrated to a protein storage buffer (50 mM Tris-HCl, 150 mM NaCl, pH 7.5) by Amicon® Ultra-30 Centrifugal Filter Devices (Merck Millipore, Germany). Concentrations of the purified protein were determined by NanoDrop 2000c (Thermo Fisher Scientifc, Waltham, MA, USA), using its extinction coefficient of 204,675 1/(mol*cm).

### Enzymatic characterization

First, 1 µM purified NFAg31A was incubated with 2 mM *p*NPαG, *p*NPβG, *p*NPαM, *p*NPβX, or *p*NPαA in phosphate buffer (pH 6.5) at 40 ℃ for 1 h to identify its possible activities. Then, the influence of pH on activity of NFAg31A was detected by incubating 0.25 µM enzyme with 2 mM *p*NPαG at 45 ℃ for 30 min between 5.5 and 9.5 (50 mM citrate buffer for pH 5.5-6.0, 50 mM phosphate buffer for pH 6.0–8.0, 50 mM Tris-HCl buffer for pH 8.0-9.5), while the influence of temperature on its activity was measured at its optimal pH for 30 min, under different temperatures ranging from 31 ℃ to 85 ℃. Unless otherwise specified, the standard activity of NFAg31A was performed by incubating 0.25 µM enzyme with 2 mM *p*NPαG at pH 7.0 and 45 ℃ for 30 min and terminating the reaction by heating at 100 ℃ for 10 min throughout this work, and the *p*-nitrophenol liberated was measured at 410 nm.

The effect of pH on stability of NFAg31A was determined by incubating 7.5 µM enzyme without substrates at room temperature for 30 min under different pH ranging from 3.0 to 12.0, and its residual activities were subsequently determined with 2 mM *p*NPαG at pH 7.0 and 45 ℃ for 30 min as described above. Meanwhile, the effect of temperature on stability of NFAg31A was measured by incubating 7.5 µM enzyme at different temperatures (from 35 ℃ to 51 ℃) for 30 min, and its residual activities were then measured in a same way. In addition, thermostability of NFAg31A at 39 ℃, 45 ℃ and 50 ℃ were detected by incubating 7.5 µM enzyme at those temperatures for different periods, and standard assay was performed to detect its residual activities.

The effects of various metal salts and chemicals on activity of NFAg31A were detected by the incubation of 0.25 µM purified enzyme with those additives at 1 mM or 10 mM concentration under standard assay, using 2 mM *p*NPαG as substrates. The activity determined in the absence of additives was taken to be 100%, and those relative activities with additives were calculated as a percentage of its activity.

### Specific activities and kinetic hydrolysis on various substrates

The specific activities of NFAmy13A were detected by incubating 0.25 µM enzymes with 2 mM *p*NPαG, 2 mM oligosaccharides (maltose, maltotriose, maltotetraose, maltopentaose, nigerose, kojibiose), and 2 mg/ml soluble starch, or incubating 4 µM enzymes with 2 mM isomaltose and sucrose, at pH 7.0 and 45 ℃ for 15 min, respectively. The reaction was terminated by heating at 100 ℃ for 10 min. Same as previous study [11], the glucose and malto-oligosaccharides liberated from nature substrates were detected by HPAEC-PAD using a Dionex CarboPac PA20 analytical column (3 × 150 mm) and Dionex CarboPac PA20 guard column (3 × 30 mm), with mobile phase of component A (250mM NaOH) and B (10mM NaOH, 500mM NaOAc), while glucose (G1), maltose (M2), maltotriose (M3), maltotetraose (M4), and maltopentaose (M5) were used as standards. Meanwhile, the *p*-nitrophenol liberated from *p*NPαG was measured at 410 nm with a molar extinction coefficient of 55,560 M^− 1^ cm^− 1^. One unit of the enzyme activity was defined as the amount of enzyme required to release 1µmol glucose equivalent from substrates per minute under its standard conditions. All reactions were performed in triplicate.

The kinetic experiments were performed by incubating the purified NFAmy13A at 45 ℃ and pH 7.0 for 15 min with increasing concentrations of substrates, i.e., 0.5–40 mM malto-oligosaccharides (maltose, maltotriose, maltotetraose and maltopentaose), 0.25–24 mM nigerose and kojibiose, 0.1–32 mM *p*NPαG, 0.5–40 mg/ml soluble starch, 2–24 mM isomaltose, respectively. The velocities were kept in a constant scope within this time frame, and low concentration of 0.25 µM enzyme was used on *p*NPαG, malto-oligosaccharides, nigerose, kojibiose and soluble starch, except for 4 µM enzyme on isomaltose. The kinetic parameters were estimated by fitting data to the Michaelis-Menten equation using a nonlinear regression method (Origin 9). To estimate kinetic parameter on soluble starch, the molar concentration was converted by assuming that the average degree of polymerization per one non-reducing end of a starch chain is 43.5 [26].

### Inhibition of monosaccharides on activity of NFAg31A

The possible inhibition of monosaccharides on activity of NFAmy13A was determined by incubation of 1 µM purified enzyme with 2 mM *p*NPαG at 45 ℃ and pH 7.0 for 30 min, containing the increasing concentrations (0.5–50 mM) of monosaccharides, i.e., glucose, mannose, xylose, fructose, galactose, arabinose. The relative activity was calculated as a percentage of the activity without the addition of monosaccharide under same condition.

### Time-course degradation of malto-oligosaccharides by NFAg31A

Time-course degradation of malto-oligosaccharides was measured by incubating 2 µM NFEg31A with high concentration of 5 mM malto-oligosaccharides at 45 ℃ and pH 7.0 for various times (5 min, 30 min, 2 h, 4 h, 8 h). Then, the glucose and malto-oligosaccharides released were detected by HPAEC-PAD under same condition as described above.

### Mimic the synergistic degradation of wheat starch in daqu system

The possible synergistic effect among NFEg31A and two previously characterized α-amylases (NFAmy13A and NFAmy13B) was studied here, since all of them were obtained from Nong-flavor daqu. Different molar concentrations and ratios were selected among them, i.e., 1:1:1 (0.5 µM NFAmy13B: 0.5 µM NFAmy13A : 0.5 µM NFEg31A), 3:7:10 (0.3 µM NFAmy13B : 0.7 µM NFAmy13A : 1 µM NFEg31A), and 1:19:20 (0.05 µM NFAmy13B : 0.95 µM NFAmy13A : 1 µM NFEg31A), so as to deeply compare their synergistic degradation of starch in daqu system. Then, different combinations of three enzymes, i.e., single enzyme (NFAmy13B, NFAmy13A, or NFEg31A), two enzyme combinations (NFAmy13B and NFAmy13A, NFAmy13A and NFEg31A, or NFAmy13B and NFEg31A), three enzyme combination (NFAmy13B, NFAmy13A and NFEg31A), were applied to the degradation of 1 mg/ml wheat starch at 45 ℃ and pH 7.0 for 30 min, respectively.

### Phylogenetic analysis of NFAg31A

NCBI-BLAST database searches were used to identify homologous α-glucosidases, and those characterized enzymes were finally selected for construction of phylogenetic tree. Accession numbers for all those selected enzymes were listed in Supplemental Table S1. Multiple sequence alignments were produced using the program ClustalW with default parameters. The phylogenetic tree was built by Mega 7 software using the Neighbour-Joining Method.

### Nucleotide sequence accession numbers

The nucleotide sequence of NFEg31A was deposited in the NCBI GenBank database with Accession Number of OP756524.

## Results

### Expression level in Nong-flavor daqu and phylogenetic analysis of NFAg31A

The ORF15963 showed high expression level with an RPKM value of 127.1, and its gene product (NFAg31A) was the 25th highest expression carbohydrate-active enzymes at N3 stage of NF daqu (Supplemental Table S2). Moreover, its gene product was the second highest expressed α-glucosidases in GH31 family at N3, and showed the highest expression level at N3 among the whole making process of NF daqu, thus indicating its pivotal and unique contribution to the starch degradation at this stage (Table S3). The ORF15963 encoded a protein with 960 amino acids (aa), and its gene product was comprised of a putative α-glucosidase domain belonging to GH31 family and DUF5100 domain (Supplemental Fig. S1a), when analyzed by the Pfam server (http://pfam.sanger.ac.uk/). According to analysis on SignalP 6.0 Server (https://services.healthtech.dtu.dk/service.php?SignalP), a signal peptide of 30 aa was predicted in its gene product, and without signal peptide, NFAg31A had a predictive molecular mass of 105.6 kDa.

**Fig. 1:**
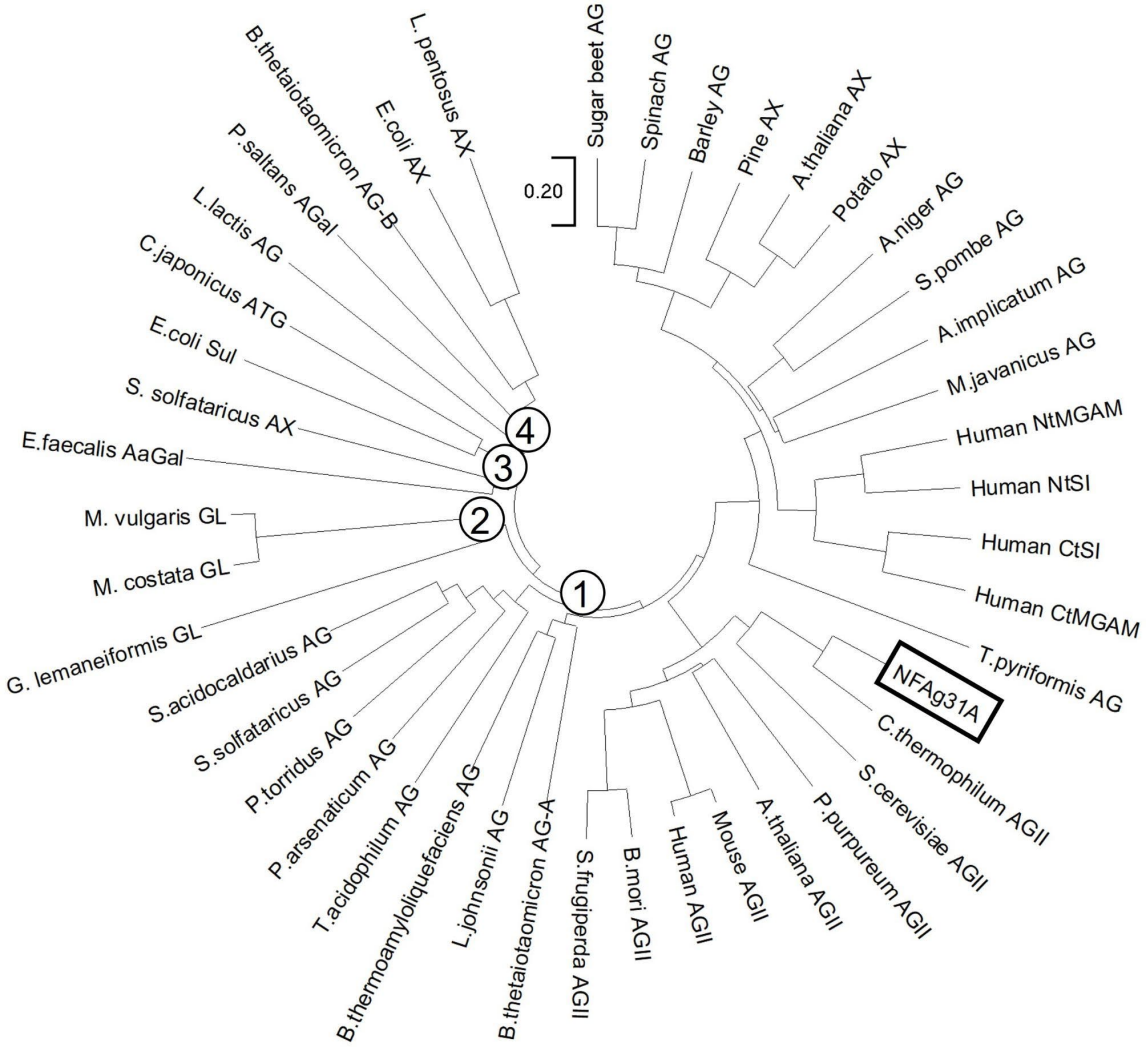
Phylogenetic tree of NFAg31A and other characterized members in four subgroups of GH31 family. Only sequences of characterized enzymes were used for analysis, which were identified as α-glucosidases (AG), maltase-glucoamylase (MGAM), sucrase-isomaltase (SI), α-glucan lyases (GL), α-transglucosylase (ATG), α-galactosidase (AGal), α-N-acetylgalactosaminidases (AaGal) and α-xylosidases (AX).

When analyzed with phylogenetic tree, enzymes in GH31 family can be mainly divided into four subfamilies, and NFAg31A was classified as a member of GH31-1 subfamily according to its conserved sequence of WNDMNE around active sites (Fig. S2), which is characteristic motif of this sub-family [27]. Those characterized enzymes in GH31-1 subfamily included all known α-glucosidase (AG), maltase-glucoamylase (MGAM), sucrase-isomaltase (SI), and α-xylosidases (AX) from plants, animals, fungi, and insects, respectively (Fig. 1). Moreover, NFAg31A was close to a group of α-glucosidase II (AGIIs).

### Cloning, expression, and purification of NFAg31A

The gene fragment of NFAg31A without signal peptide was directly amplified from N3 cDNA, cloned into pET28-a + and successfully expressed in *E.coli* BL21(DE3) as intracellular soluble form. Without C-terminal DUF5100 domain, NFAg31A expressed as intracellular insoluble form (data not shown). Then, the recombinant NFAg31A was purified by affinity chromatography using Ni^2+^-NTA resin. The purified NFAg31A showed a clear band at above 100 kDa, in good agreement with its predictive value (105.6 kDa) (Fig. S1b).

### Enzymatic characterization of NFAg31A

**Fig. 2:**
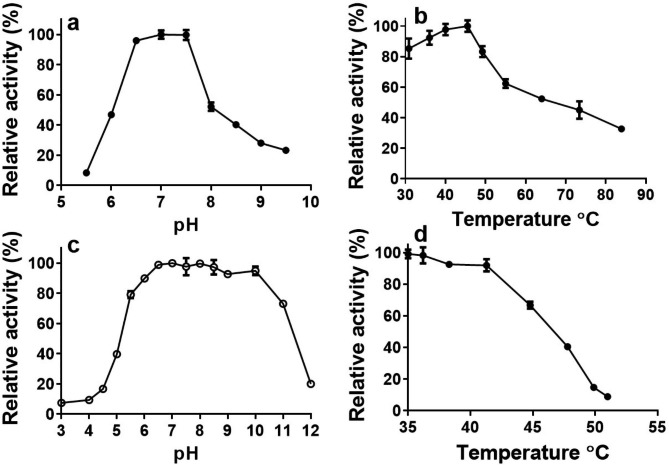
Effects of pH and temperature on activity and stability of NFAg31A. The effects of pH (**a**) and temperature (**b**) on activity of NFAg31A were determined by incubating enzyme with *p*NPG in different pHs (pH 5.5 to pH 9.0) and various temperatures (31 ℃ to 85 ℃) for 30 min, respectively. While effects of pH (**c**) and temperature (**d**) on stability of NFAg31A were detected by preincubating enzyme for 30 min under different pH ranging from 3.0 to 12.0, or at different temperatures (from 35 ℃ to 51 ℃), respectively, and their residual activities were subsequentially determined at standard condition. Each experiment was performed in triplicate

The purified NFAg31A only showed clear activity on *p*NPαG, not on *p*NPβG, *p*NPβX, *p*NPαM, or *p*NPαA (data not showed), indicating the α-glucosidase activity of NFAg31A. As shown in Fig. 2a and b, NFAg31A showed an optimal activity at pH 7.0 and 45 ℃, and it exhibited good stability over a broad pH ranging from pH 6.0 to pH 10.0, keeping more than 90% residual activity (Fig. 2c). Meanwhile, NFAg31A was stable up to 41.3 ℃ for 30 min, with ~ 92% residual activity (Fig. 2d). Furthermore, it kept more than 60% activity at 39 ℃ for 180 min, still kept more than 50% activity at 45 ℃ for 60 min, and nearly lost 80% activity at 50 ℃ within 10 min (Fig. S3). Additionally, at 1 mM concentration, none of those detected additives showed stimulation on activity of NFAg31A, except for a slight enhancement by Ba^2 +^ and Mg^2+^, while a clear inhibition was observed for most of those detected additives, such as slight inhibition of Ca^2+^, Ni^2+^, Al^3+^, K^+^ and Li^+^, moderate inhibition of Al^3+^, Mn^2+^, EDTA·Na_2_ and NH_4_^+^, severe inhibition of Zn^2+^, Hg^2+^ and SDS, and complete inhibition of Fe^3+^ and Cu^2+^ (Table S4). Whereas, all of those additives showed an increasing inhibition at high concentration of 10 mM compared with that at 1 mM.

The specific activity was examined with glucosidic substrates having all possible types of α-linkages, including oligosaccharides and starch. As shown in Table 1, NFAg31A exhibited a broad specificity on the linkages of α-glycosidic bond. Higher activity (1.59 U/mg) was observed on nigerose (α-1,3 bond) than on other di-saccharides, i.e., maltose (α-1,4 bond), kojibiose (α-1,2 bond), isomaltose (α-1,6 bond). But activity on isomaltose (0.0017 U/mg) was very weak. Whereas NFAg31A could hydrolyze all the detected maltosaccharides, with the specific activity ranging from 0.24 to 0.82 U/mg, and showed significantly higher activities on maltotriose and maltotetraose than on maltose and maltopentose. More importantly, NFAg31A could also directly hydrolyze soluble starch into maltosaccharides, with the highest specific activity of 2.40 U/mg among all detected substrates, which to some extent might indicate its preference on longer malto-oligosaccharide. In contrast, NFAg31A showed no activity on sucrose, which is comprised of glucose and fructose with α-1,2 linkage.

**Table 1:** The kinetic hydrolysis of various substrates by NFAg31A

Substrate	Main linkage/monomer	*K*_m_(mM)	*k*_cat_(1/s)	*k*_cat_/*K*_m_(1/(s*mM))	Specific activity(U/mg)
*p*NPαG	α-(1→4)-glucose	0.55 ± 0.06	0.10 ± 0.00	0.19	0.053 ± 0.001
Maltose	α-(1→4)-glucose	11.5 ± 0.7	4.4 ± 0.1	0.39	0.38 ± 0.01
Maltotriose	α-(1→4)-glucose	nd^a^	nd^a^	0.59 ± 0.04^b^	0.83 ± 0.00
Maltotetraose	α-(1→4)-glucose	nd^a^	nd^a^	0.85 ± 0.04^b^	0.60 ± 0.06
Maltopentaose	α-(1→4)-glucose	nd^a^	nd^a^	0.37 ± 0.03^b^	0.24 ± 0.02
Soluble Starch	α-(1→4)-α-(1→6) glucose	nd^a^	nd^a^	14.2 ± 0.6^b^	2.40 ± 0.24
Nigerose	α-(1→3)-glucose	19.3 ± 2.5	29.3 ± 2.1	1.52	1.59 ± 0.02
Kojibiose	α-(1→2)-glucose	3.9 ± 0.3	3.2 ± 0.1	0.82	0.63 ± 0.03
Isomaltose	α-(1→6)-glucose	nd^a^	nd^a^	0.0014 ± 0.0001^b^	0.0017 ± 0.0001
Sucrose	glucose-α-(1→2)-fructose	-	-	-	No activity

^a^: The kinetic hydrolysis of those substrates did not fit very well to the Michaelis-Menten equation and the enzyme was not saturated with the substrate under detected concentrations, thus their kinetic values did not determine here

^b^: The *k*_cat_/*K*m values were determined at sufficiently low substrate concentration, by the relationship *k*_cat_/*K*m = v/([S][E]), which is valid for [S] < < *K*m.

Since NFAg31A was not saturated with some substrates, i.e., maltotriose, maltotetraose, maltopentose, soluble starch and isomaltose, and their kinetic hydrolysis did not fit well to Michaelis-Menten equation, their kinetic values (*K*_m_ and *k*_cat_) were not determined and only *k*_cat_/*K*_m_ values were determined at sufficiently low substrate concentration (Table 1). Similar as the trend of specific activity, soluble starch demonstrated the highest catalytic efficiency *(k*_cat_/*K*_*m*_) of 14.2 1/(s*mM), and isomaltose displayed the lowest *k*_cat_/*K*_*m*_ value of 0.0014 1/(s*mM). Furthermore, among di-saccharides, kojibiose showed the lowest *K*_m_ value of 3.9 mM, which could indicate a strong binding affinity between NFAg31A and kojibiose. Meanwhile, NFAg31A displayed a dramatically higher *k*_cat_ value of 29.3 1/s on nigerose, which was 6.7 and 9.2 folds of that on maltose and kojibiose, respectively, which would be the main reason that NFAg31A exhibited higher catalytic efficiency on nigerose than on kojibiose or maltose. In addition, *p*NPαG, the unnatural substrate, displayed a relatively low kinetic values and specific activity, when compared to oligosaccharides and starch, except isomaltose (Table 1).

### Inhibition of monosaccharides on activity of NFAg31A

**Fig. 3:**
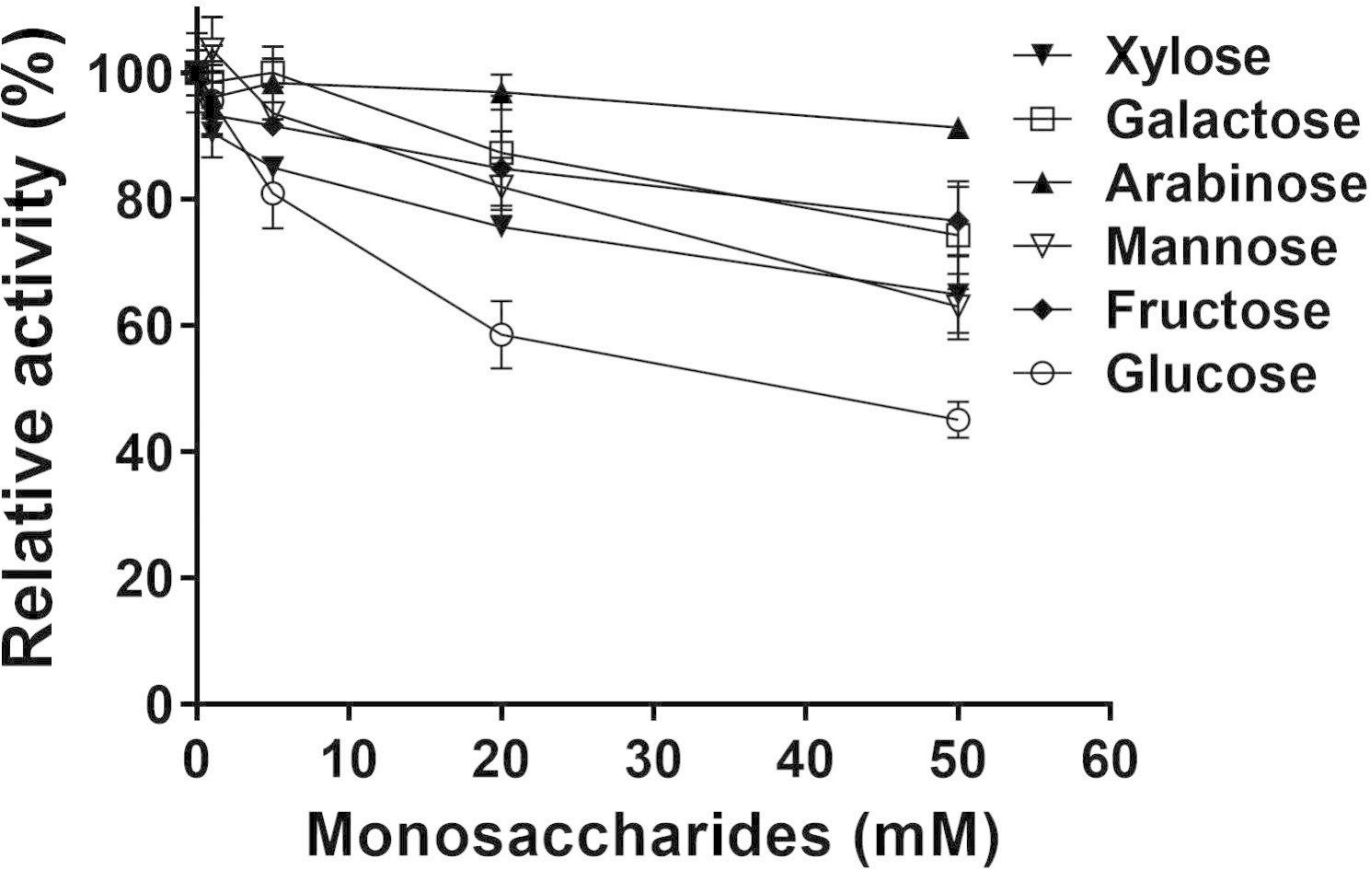
Inhibitions of monosaccharides on activity of NFEg31A. Experiments were determined by incubation of enzyme with the increasing concentrations (0.5–50 mM) of monosaccharides under standard condition. Each experiment was performed in triplicate

As shown in Fig. 3, all of those detected monosaccharides showed an obvious inhibition, decreasing the activity of NFAg31A with an increase of monosaccharide concentration. Among them, glucose showed the largest inhibition, decreasing around 55% activity of NFAg31A at 50 mM concentration, arabinose showed the smallest inhibition, only decreasing less than 10% activity at the same concentration, and the inhibition of galactose, fructose, mannose, and xylose was in middle at all measured concentrations.

### Time-course degradation of malto-oligosaccharides by NFAg31A

**Fig. 4:**
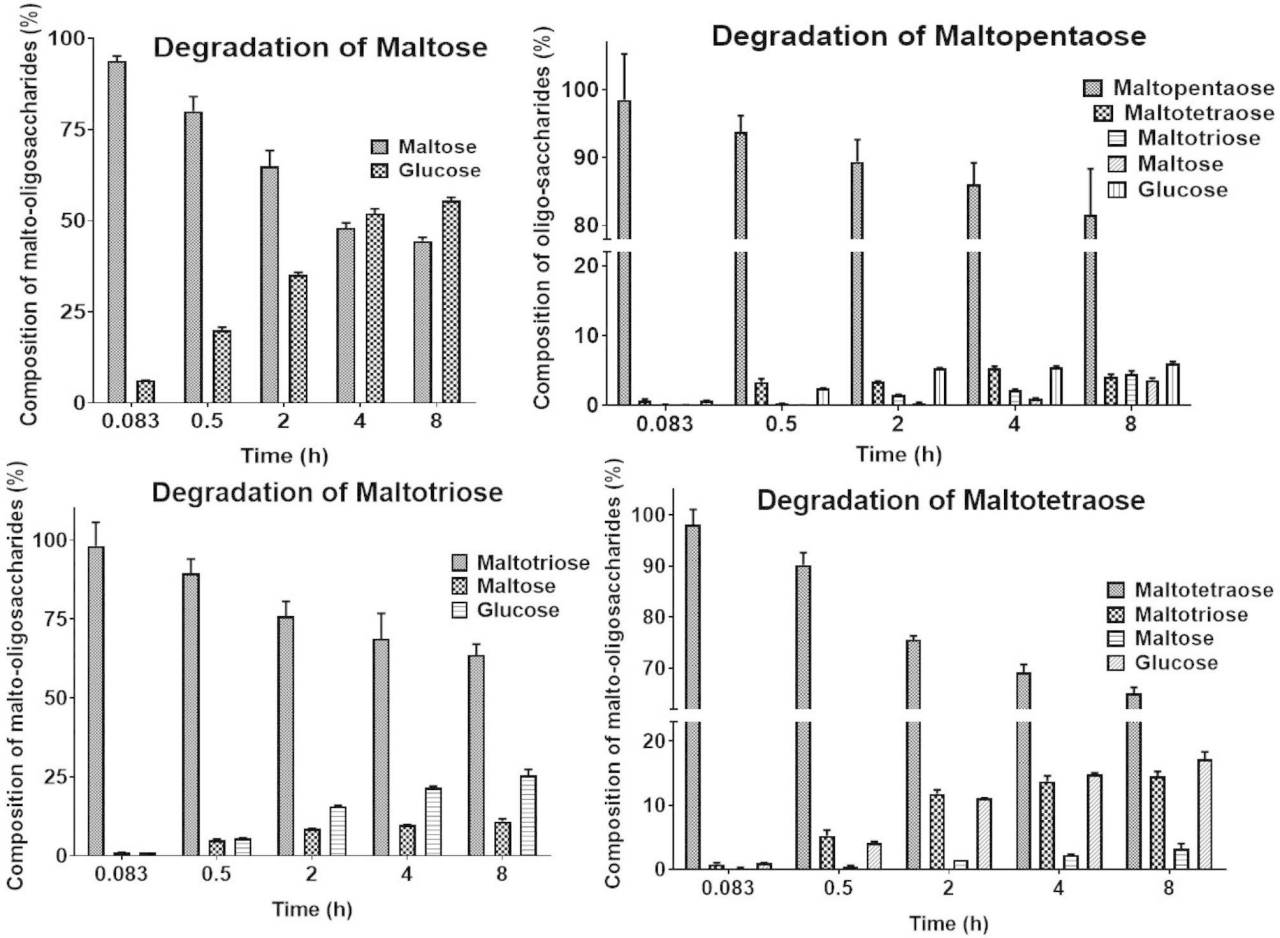
Degradation of Malto-oligosaccharides by NFEg31A. Degradations were detected by incubating high concentration of 2 µM NFEg31A with 5mM malto-oligosaccharides under standard condition for various times, and their end products were analyzed with HPAEC-PAD. Each experiment was performed in triplicate

To get close to the actual degradation of malto-oligosaccharides by NFAg31A in NF daqu, 2 µM NFEg31A was applied in degrading high concentration of 5 mM malto-oligosaccharides for various times. As shown in Fig. 4, the degradation of malto-oligosaccharides all increased with the increase of time and reached the highest degradation at 8 h. Interestingly, NFEg31A could release more products of glucose from maltose under all time periods, being more efficient to hydrolyze maltose (M2) than longer malto-oligosaccharides. Equal amount of glucose (G1) and M2 was released from maltotriose (M3) within half an hour, thereafter, since M2 would be further hydrolyzed into G1, more glucose was produced than maltose, with around 11% M2 and 25% G1 at 8 h. Meanwhile, the main products from maltotetraose (M4) were G1 and M3 throughout the incubation times, which should indicate its preference on cleaving M4 into G1 and M3. Additionally, maltopentose (M5) were mainly hydrolyzed into G1 and M4 within the incubation period of 4 h, which might indicate its preference on converting M5 into G1 and M4.

### Mimic the synergistic degradation of starch in NF daqu

**Fig. 5:**
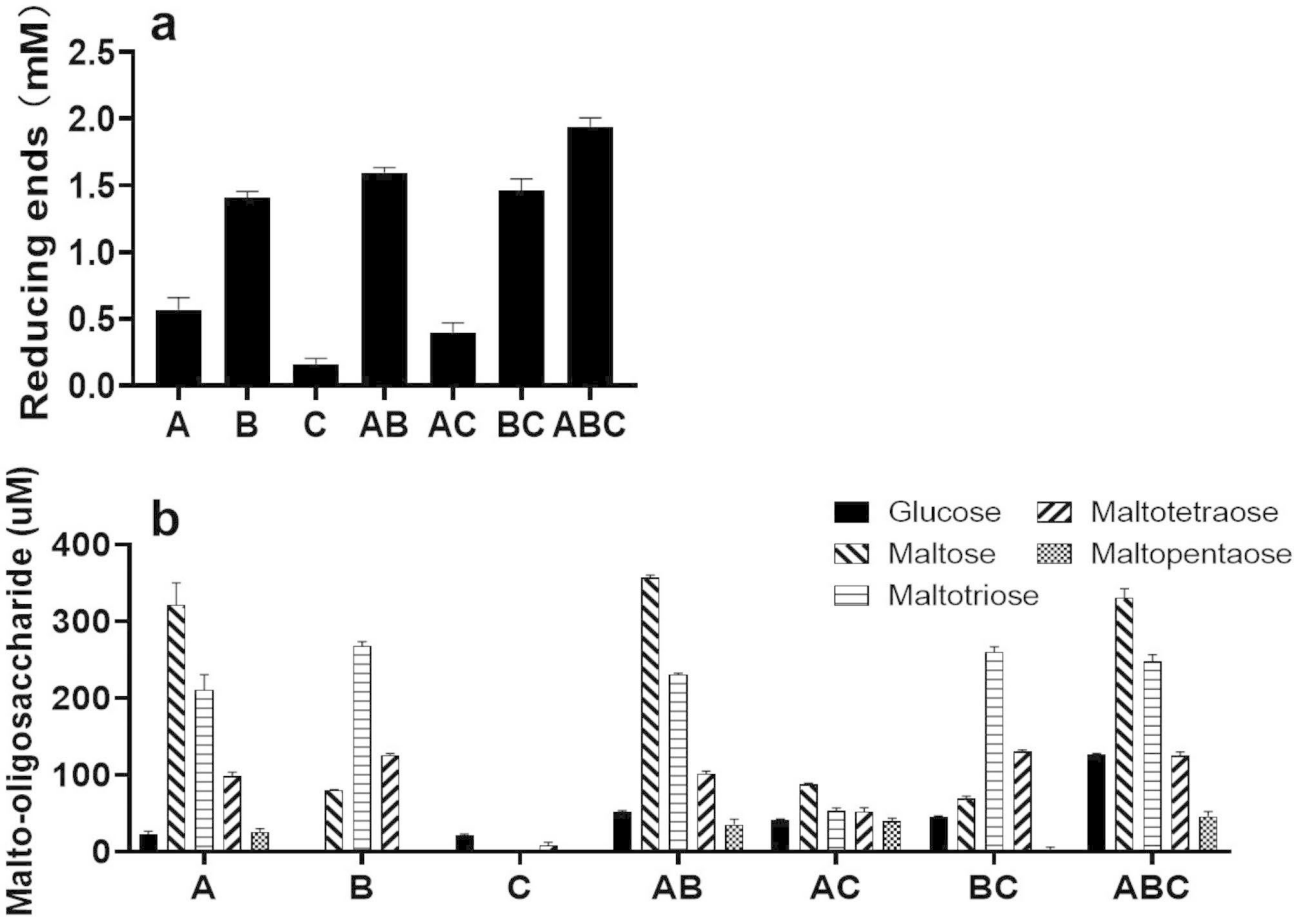
Synergistic hydrolysis of NFAmy13A, NFAmy13B and NFAg31A on starch. Same molar ratio of NFAmy13B:NFAmy13A:NFAg31A (3:7:10) and their different combinations were used to hydrolyze starch here. The reducing products (**a**) were analyzed with *p*HBAH method, and end products (**b**) were analyzed with HPAEC-PAD. A: NFAmy13A; B: NFAmy13B; C: NFAg31A; AB: NFAmy13A + NFAmy13B; AC: NFAmy13A + NFAg31A; BC: NFAmy13B + NFAg31A; ABC: NFAmy13A + NFAmy13B + NFAg31A. Each experiment was performed in triplicate

Under same condition, more reducing products (2.70 mM) were released from starch by NFAmy13B than 0.61 mM by NFAmy13A (Table S5), and the lowest hydrolyzing capacity on starch was observed for NFAg31A, releasing the smallest amount (0.07 mM) of reducing products. When molar ratio of 1:1:1 and 1:19:20 were applied, synergistic effect was not obviously observed between NFAg31A with NFAmy13B or with NFAmy13A, or among three enzymes. Only when molar ratio of 3:7:10, namely 0.3 µM NFAmy13B: 0.7 µM NFAmy13A: 1.0 µM NFAg31A, was used, a significantly synergistic effect was identified among three enzymes. In detail, three enzyme combination, as well as two enzyme combinations, definitely released more reducing products from soluble starch than those from the related single enzymes (Fig. 5 and Table S5).

Strangely, NFAmy13B released higher amount (1.40 mM) of reducing products than that (0.57 mM) of NFAmy13A (Fig. 5a and Table S5), but it only released higher values of maltotriose and maltotetraose than NFAmy13A, and its total end products, as well as amount of maltose and glucose, were all lower than those of NFAmy13A (Fig. 5b and Table S6), indicating that a larger number of longer malto-oligosaccharides (> M5), which could not be efficiently detected by present HPAEC-PAD method, were produced by NFAmy13B than that by NFAmy13A. Furthermore, when NFAmy13A and NFAmy13B were combined, each amount of end products (G1 to M5) increased. Since both of NFAmy13A and NFAmy13B could not degrade maltose, the addition of NFAg31A to NFAmy13A or NFAmy13B definitely increased the glucose products. In addition, NFAg31A might have a competitively synergistic effect with NFAmy13A, since their combination released lower amount of end products, except for glucose, than that by single NFAmy13A (Fig. 5b and Table S6). More importantly, three enzyme combination largely increased the amount of each malto-oligosaccharide, especially the content of glucose, compared to any enzyme combinations and single enzymes. Therefore, it was reasonable to conclude that NFAg31A showed a competitive and synergistic effect with NFAmy13A and NFAmy13B.

## Discussion

Plenty of microbial community was openly enriched in Chinese NF Daqu, and the highest number of 932 carbohydrate-active enzymes were detected at its high-temperature stage by metatranscriptomics [9]. Since the main component of NF daqu is starch, the starch degrading enzymes definitely play the largest contribution to the quality of NF Daqu. Thus, two α-amylases (NFAmy13 and NFAmy13B) were sequentially obtained by a metatranscriptomics-based method from NF Daqu. Here, a novel α-glucosidase was similarly mined, thereafter its enzymatic characterization, as well as its synergistic effect with NFAmy13A and NFAmy13B, was thoroughly studied, so as to comprehensively shed light on the complicate degradation of starch in NF daqu.

The gene products of ORF 15,963 was predicted to encode a signal peptide of 30aa in front of the mature protein (NFAg31A), and without this signal peptide, NFAg31A was successfully obtained with active form by heterologous expression in *E.coli* BL21(DE3). A similar signal peptide with same size (30 aa) and high similarities (> 70%) was also found in putative AGIIs from fungal species, such as *Aspergillus clavatus* (XP_001276182.1), *Penicillium manginii* (KAJ5741752.1), *Penicillium cataractarum* (KAJ5381475.1) and *Aspergillus avenaceus* (KAE8150115.1). And without the same size peptide, *C.thermophilum* AGII was also successfully obtained by heterologous expression in *E. coli* BL21-CodonPlus (DE3) and folded correctly [28]. Thus, this finding might indicate that NFAg31A have a similar signal peptide, as well as secretory pathway, with those fungal AGIIs. Moreover, the amino acid sequence of NFAg31A exhibited the highest similarity (85.2%) with putative α-glucosidases (XP_028489865.1) from *Paecilomyces variotii*, and the highest similarity (65.8%) with characterized AGII from *C.thermophilum* [14], indicating that NFAg31A might also come from a fungal species. Meanwhile, when analyzed by phylogenetic tree, together with *C.thermophilum* AGII and *Saccharomyces cerevisiae* AGII, NFAg31A was clustered to a group of α-glucosidases II (AGIIs), and NFAg31A showed a similar molecular size (105.6 kDa) with its homologous AGIIs, within size ranges from 98 kDa to 125 kDa [14, 18, 29–31].

NFAg31A showed optimal activity at 45 ℃, which was similarly detected as 45 ℃ of *Apis cerana* AGII [32] and 50 ℃ of *Acremonium implicatum* AG [33], litter higher than that (35 ℃ − 37 ℃) of many homologous AGs from animals, fungi, plants, insects and bacteria [13, 18, 28–30, 32, 34–37], but pretty lower than that (80 ℃ − 100 ℃) of archaebacterial α-glucosidases [15, 38–40]. In addition, NFAg31A was a mesophilic α-glucosidase, being stable up to 41.3 ℃, which is litter higher than 38 ℃ of *Lactobacillus johnsonii* AG [26] and 40 ℃ of *Schizosaccharomyces pombe* AGII [34], and lower than 55 ℃ of *A.cerana* AGII [32]. Whereas, NFAg31A showed an optimal pH at ~ 7.0, which was similar as pH 6.5 to pH 7.5 for most of homologous AGIIs [16, 18, 29, 30, 41], AGs [13, 42], and human MGAM and SI [22, 43, 44], different with pH 3.5 to 5.5 for *A.cerana* AGII and archaebacterial AGs. Moreover, NFAg31A was a neutral α-glucosidase, exhibiting well stability over a broad pH range of pH 6.0 to pH 10.0, which was similarly detected as pH 6.2 to pH 9.1 for *S.pombe* AGII and pH 5.0 to pH 8.0 for *L.johnsonii* AG. Therefore, NFAg31A showed similar enzymatic characterization over temperature and pH as its close members in GH31-1 subfamily, e.g., *A.implicatum* AG and *S.pombe* AGII. In short, based on both of its sequnce alignment and enzymatic characterization analysis, NFAg31A should be a typic fungal member in GH31-1 subfamily.

Meanwhile, same as its close member of *S.cerevisiae* AGII in GH31-1 subfamily [45], the activity of NFAg31A was not clearly stimulated by any of those detected metal ions and chemicals, indicating that NFAg31A might be not a metalloenzyme, and on the contrary, activities of bacterial members of *Sulfolobus acidocaldarius* AG [38] and *Thermoplasma acidophilum* AG [39] were both enhanced by 1mM Mn^2+^. Furthermore, its activity was strongly inhibited by SDS, Cu^2+^, and Zn^2+^, which was same as that of *S.acidocaldarius* AG and *T.acidophilum* AG.

As shown in Table 2, NFAg31A showed efficient degradability on homogenous substrates, e.g., malto-oligosaccharides, soluble starch, kojibiose and nigerose, with higher activity on starch than malto-oligosaccharides, and showed little or no activity to heterogeneous substrate such as sucrose and *p*NPαG, indicating that it should be a type III AG [16]. Meanwhile, it could efficiently degrade various α-glycosidic substrates, with comparable capacities with that of AGIIs from *A.cerana*, *Sporothrix schenckii* and *S.pombe*, human MGAM and SI, and AGs from *L.johnsonii*, *Lactococcus lactis*, *S.solfataricus* and *Pyrobaculum arsenaticum*. Moreover, same as its homologous α-glucosidases II (AGIIs) from *C.thermophilum* [14], *S.cerevisiae* [18], *Porphyridium* sp. (Rhodophyta) [29], *Bombyx mori* and *Spodoptera frugiperda* [30], NFAg31A showed the highest activity on nigerose (Glc-α1,3-Glc) among di-saccharides, and similar preference on Glc-α1,3-Glc was also detected for other α-glucosidases from *L.lactis*, *A.implicatum* and *L.johnsonii* (Table 2). Besides this preference, it exhibited lower but comparable activities on Glc-α1,2-Glc and Glc-α1,4-Glc, while efficient capacities on hydrolyzing three glycosidic linkages were only reported for *Sulfolobus acidocaldarius* AG and *T.acidophilum* AG. During hydrolysis, di-saccharides would bind the catalytic pocket of α-glucosidases by interaction of its nonreducing terminal Glc with the residues of subsite − 1 and the reducing Glc with the residues of subsite + 1 [13, 14]. For NFAg31A, those residues were Asp401, Ile438, Asp440, Trp475, Trp512, Trp588, Asp620, His649 at subsite − 1, and Asp261, Arg575, Phe624 at subsite + 1, as well as Asp514 and Asp591 at its catalytic sites, which were highly conserved in most of its homologous AGs, including *S.acidocaldarius* AG and *T.acidophilum* AG (Fig. S2). LlAG kept different residues at sites of Asp261, Ile438, Asp440, Trp475, Phe624 (Fig. S2), which could be the key reason that it showed strict specificity on Glc-α1,3-Glc, and pretty low activity on Glc-α1,4-Glc [13], when compared with NFAg31A, *S.acidocaldarius* AG and *T.acidophilum* AG. Besides those high activities on Glc-α1,3-Glc, Glc-α1,2-Glc and Glc-α1,4-Glc, *S.acidocaldarius* AG and *T.acidophilum* AG still showed relative high activities on Glc-α1,6-Glc (Table 2), whereas, NFAg31A showed pretty low activity on Glc-α1,6-Glc, which might result from their differences around those conserved residues at subsite − 1 and subsite + 1 (Fig. S2). Thus, NFAg31A would efficiently recognize Glc-α1,3-Glc, Glc-α1,2-Glc and Glc-α1,4-Glc by those conserved residues at subsite − 1 and subsite + 1 *via* hydrogen bonds and hydrophobic interaction [13, 14], which could be mainly responsible for its substrate specificity, and more mutations at those sites would be done to get close to the specific function of each of residues in future.

**Table 2:** Comparison of hydrolyzing capacities among NFAg31A and α-glucosidases in GH31-1 subfamily

Enzyme	Origin	Specific activity (U/mg)	Kcat/Km	The degrading patterns	References
NFAg31A	Fungi from NF daqu	1.59(nigerose); 0.63(kojibiose); 0.38(maltose); 2.40(soluble starch); 0.0017(isomaltose); 0.053 ± 0.001(pNPαG)	1.52(nigerose); 0.82(kojibiose); 0.39(maltose); 14.9(soluble starch) 0.0014(isomaltose); 0.19(pNPαG)	Glc-α1,3-Glc > Glc-α1,2-Glc > Glc-α1,4-Glc > > Glc-α1,6-Glc	This study
AcAGII	*Apis cerana*	0.0016(*p*NPαG)	-	Glc-α1,3-Glc > Glc-α1,3-Man	[32]
SsAGII	*Sporothrix schenckii*	0.19(nigerose); 0.06(kojibiose); 0.05(maltose); 0.02(isomaltose); 0.46(4-methylumbelliferyl-a-D-glucopyranoside)	-	Glc-α1,3-Glc > Glc-α1,3-Man	[41]
SpAGII	*Schizosaccharomyces pombe*	-	0.048(nigerose); 0.003(kojibiose); 0.013(maltose); 0.00001 (isomaltose); 0.004(*p*NPαG)	Glc-α1,3-Glc > Glc-α1,3-Man	[34]
NtMGAM	Human	7.57(maltose); 0.10(isomaltose)	-	Glc-α1,4-Glc > > Glc-α1,6-Glc	[21]
NtSI	Human	-	19(maltose); 9(isomaltose)	Glc-α1,4-Glc > Glc-α1,6-Glc	[44]
CtMGAM	Human	-	3.97(maltose)	Glc-α1,4-Glc	[22]
SbAG	*Sugar beet*	-	13.7(maltose); 1230(soluble starch)	Glc-α1,4-Glc	[37]
AiAG	*Acremonium implicatum*	-	732(nigerose);286(maltose); 10.8 (kojibiose); 1280(soluble starch)	Glc-α1,3-Glc > Glc-α1,4-Glc > Glc-α1,2-Glc	[33]
AnAG	*Aspergillus niger*	-	*558*(maltose); 188(soluble starch)	Glc-α1,4-Glc	[37]
LjAG	*Lactobacillus johnsonii*	-	43.9(nigerose); 26.1(kojibiose); 3.99(maltose); 10.6(isomaltose); 0.712(soluble starch)	Glc-α1,3-Glc, Glu-α-1,4-Fru and Glc-α1,2-Glc > Glc-α1,6-Glc > Glc-α1,4-Glc, Glu-α-1,6-Fru, Glu-α-1,3-Fru and Glu-α-1,5-Fru	[26]
TaAG	*Thermoplasma acidophilum*	582(maltose); 445(kojibiose); *233*(nigerose); 153(isomaltose);	220(maltose); 103(kojibiose); 5.4(isomaltose);	Glc-α1,4-Glc > Glc-α1,2-Glc > Glc-α1,3-Glc > > Glc-α1,6-Glc	[39]
LlAG	*Lactococcus lactis*	*6.3*(nigerose);*0.34*(kojibiose); *0.01*(maltose); 0.003(isomaltose);	1.1(nigerose);0.1(kojibiose); 0.02(maltose);	Glc-α1,3-Glc > > Glc-α1,2-Glc > > Glc-α1,4-Glc > Glc-α1,6-Glc	[13]
SsAG	*Sulfolobus solfataricus*	3.32(maltose);0.25(isomaltose); <0.01(starch)	-	Glc-α1,4-Glc > Glc-α1,6-Glc	[49]
SaAG	*Sulfolobus acidocaldarius*	*799.2*(maltose); 578.6(kojibiose); 193.7(nigerose);156.3(isomaltose); 4.4(starch)	-	Glc-α1,4-Glc > Glc-α1,2-Glc > Glc-α1,3-Glc > Glc-α1,6-Glc	[38]
PaAG	*Pyrobaculum arsenaticum*	*5.84*(maltose); 1.53(isomaltose); 4.86(kojibiose); 5.24(nigerose)	-	Glc-α1,4-Glc, Glc-α1,3-Glc and Glc-α1,2-Glc > Glc-α1,6-Glc	[15]

Most importantly, NFAg31A showed definitely higher degrading capacity on long-chain starch than on short-chain oligosaccharides, which was same observed for Sugar beet AG and *A.implicatum* AG, completely different with *S.acidocaldarius* AG, *A.niger* AG, *L.johnsonii* AG and *S.solfataricus* AG. The similar preference of human CtMGAM for longer oligosaccharides was speculated by an extra 21 amino acids in human CtMGAM and CtSI [22], which might form subsites + 2 and + 3, and was not found in NFAg31A (Fig. S2). Additionally, a phenylalanine residue in the loop 7 of the (β/α)_8_ domain of CtMGAM was likely responsible for this preference [21], which was conserved in NFAg31A as Phe625 (Fig. S2). Whereas, key aromatic residues of Sugar beet AG (SbAG) was proved to be associated with recognition for long-chain substrates [37], which was conserved as Tyr265 in NFAg31A (Fig. S2). Thus, these conserved aromatic residues of Phe625 and Tyr265 might indicate similar machinery for recognizing long substrates in NFAg31A.

The addition of monosaccharides definitely reduced the activity of NFAg31A, especially glucose. Indeed, glucose was also reported to largely inhibit the activity of AGII from *Porphyridium purpureum* [29] and *Candida albicans* [46]. Whereas, the catalytic nucleophile and acid/base of NFAg31A were Asp514 and Asp591, which was highly conserved among NFAg31A and its homologous AGs (Fig. S2). Therefore, it is rational to conclude that NFAg31A could be a retaining α-glycosidase in GH31 family [18, 47], which cleaves terminal carbohydrate moiety involving a covalent glycosyl-enzyme intermediate [47]. It might be further suggested that the addition of glucose or other monosaccharides cause a reverse reaction instead of hydrolysis, thus resulting in the decrease of its activity [29]. Meanwhile, monosaccharides might also act as competitive inhibitors to α-glycosidase [48], thus it is not surprising that glucose, which is the hydrolyzing target in the α-glycosidic substrates, exhibited the highest inhibitions among all detected monosaccharides.

The hydrolyzing pattern of NFAg31A was identified by time-course degradation of malto-oligosaccharides. Within short incubation of 1 h, NFAg31A definitely showed preference on removing glucose from all malto-oligosaccharides (M2 to M5). This finding again proved that NFAg31A is a typical GH31-type α-glucosidase, which could hydrolyze malto-oligosaccharides in an exo-acting manner, efficiently trimming glucose moiety from the non-reducing end of malto-oligosaccharides [13–15]. As mentioned above, when 0.25 µM enzyme was incubated with 2 mM oligosaccharides for 15 min, NFAg31A showed higher specific activity and catalytic efficiency on maltotriose and maltotetraose than on maltose and maltopentose. Strangely, when higher concentration of 2 µM NFEg31A and 5 mM malto-oligosaccharides were incubated for various times, NFAg31A clearly showed the highest degrading efficiency on maltose, followed by maltotriose and maltotetraose, and the lowest on maltopentose during the whole degrading process, especially in longer incubation times (> 30 min). One main reason for this strange finding would be severe and different glucose inhibition existing in hydrolyzing malto-oligosaccharides, since more glucose was produced in time-course degradation than that in specific and kinetic assays. More importantly, NF daqu was made in solid-state environment, in which high concentration of glucose could be accumulated around enzymes, resulting in severe glucose inhibition. Thus, NFAg31A might actually show a displaceable advantage on cleaving short-chain malto-oligosaccharides, not long-chain starch, and synergistically function with other starch degrading enzymes in NF daqu.

Previously, NFAmy13A and NFAmy13B, from same stage of Nong-flavor daqu, have showed synergistic effect in hydrolyzing starch. Here, a dramatically synergistic effect was further observed among NFEg31A, NFAmy13A and NFAmy13B in starch degradation, according to both reducing products and end products, which might result from their different hydrolyzing patterns [17]. In detail, it was again proved previous finding that NFAmy13B dominated the role in hydrolyzing starch into longer malto-oligosaccharides (> M5), subsequently NFAmy13A played main contribution to the hydrolysis of long-chain malto-oligosaccharides into glucose and maltose [11]. Although NFEg31A was less competent with NFAmy13A and NFAmy13B in hydrolyzing long malto-oligosaccharides (> M5) and starch, it could efficiently degrade short-chain malto-oligosaccharides (M2-M5) and showed competent effect with NFAmy13A in degrading M2-M5. Most importantly, NFEg31A could alleviate their product-inhibitions and play the irreplaceable role in cleaving maltose to glucose. Thereafter, it is reasonable to speculate that, under solid-state fermentation of NF daqu, NFAmy13B pose the key role in degrading starch into long malto-oligosaccharides (> M5), subsequentially NFAmy13A shows the main contribution to degrade those long malto-oligosaccharides into short malto-oligosaccharides (M2-M5), then NFAmy13A and NFEg31A have competent effects in hydrolyzing short malto-oligosaccharides into maltose, and finally NFEg31A plays the unique function in converting maltose into glucose. In conclusion, three enzymes all could efficiently degrade starch and malto-oligosaccharides, and they played complementary, competent and unique role in starch degradation of NF daqu under solid-state environment.

Importantly, α-glucosidase is the most abundant enzymes involved starch degradation in NF daqu and Jiang-flavor daqu [6]. Moreover, α-glucosidases were highly active in NF daqu, and NFEg31A was the second highest expressed α-glucosidases at high temperature stage of NF daqu. Therefore, taking into account their efficiently synergistic effects, NFEg31A, together with NFAmy13A and NFAmy13B, provide good opportunity for strengthening the starch-degrading capacity of NF Daqu, and in future, more work would be done to promote their actual application in NF Daqu, as well as in other traditional industries accompanying solid-state starch degradation.

## Conclusion

In present study, a fungal α-glucosidase in GH31-1 subfamily, with relatively high expression in NF daqu, was successfully obtained and identified with high catalytic efficiencies towards α-1,3-, α-1,4-, and α-1,2-glycosidic linkages. Its synergistic and irreplaceable contribution to the degradation of starch in NF daqu was also confirmed. NF daqu is a complicated enzyme system, which is enriched with huge amounts of enzymes in hydrolyzing various α-glycosidic substrates. Therefore, the broad substrate specificity and well synergistic function of NFAg31A provide chance for enhancing the degrading capacity of NF daqu, and improving the quality and production of NF baijiu.

## Electronic supplementary material

Below is the link to the electronic supplementary material.

Supplementary Material 1: **Table S1.** Accession numbers of all those selected enzymes for Phylogenetic tree analysis in four subgroups of GH31 family. **Table S2.** The top 30 highest expression of genes related to carbohydrate-active enzymes at the highest temperature stage (N3) of NF daqu. **Table S3.** The relative high expression of alpha-glucosidase (GH31) genes at the high temperature stage (N3), and their relative expressions at the beginning stage (N1), the increasing temperature stage (N2) and mature stage (N4) of NF daqu. **Table S4.** Effect of metal ions and chemicals on the activity of NFAg31A. **Table S5.** The synergistic hydrolysis of soluble starch by different molar ratios and combinations of NFAg31A and two alpha-amylases (NFAmy13A and NFAmy13B). **Table S6.** The end products of synergistically hydrolyzing soluble starch by NFAg31A and two alpha-amylases (NFAmy13A and NFAmy13B) at molar ratio of 3:7:10. **Figure S1.** Schematic representation and purification of NFAg31A. **Figure S2.** Sequence alignment among NFAg31A and its homologous ?-glucosidases. **Figure S3.** Thermostability of NFEg31A at 39 ℃, 45 ℃ and 50 ℃

## Data Availability

All the data analyzed during this study have been included in this published article and supplemental data.
