# Supplementary material for: Insight into broad substrate specificity and synergistic contribution of a fungal α-glucosidase in Chinese Nong-flavor daqu
Source: Microb Cell Fact. 2023 Jun 15;22:114. doi: 10.1186/s12934-023-02124-z (PMC10268404; doi:10.1186/s12934-023-02124-z)
Supplement: Supplementary file 1 — Supplementary Material 1: Table S1. Accession numbers of all those selected enzymes for Phylogenetic tree analysis in four subgroups of GH31 family. Table S2. The top 30 highest expression of genes related to carbohydrate-active enzymes at the highest temperature stage (N3) of NF daqu. Table S3. The relative high expression of alpha-glucosidase (GH31) genes at the high temperature stage (N3), and their relative expressions at the beginning stage (N1), the increasing temperature stage (N2) and mature stage (N4) of NF daqu. Table S4. Effect of metal ions and chemicals on the activity of NFAg31A. Table S5. The synergistic hydrolysis of soluble starch by different molar ratios and combinations of NFAg31A and two alpha-amylases (NFAmy13A and NFAmy13B). Table S6. The end products of synergistically hydrolyzing soluble starch by NFAg31A and two alpha-amylases (NFAmy13A and NFAmy13B) at molar ratio of 3:7:10. Figure S1. Schematic representation and purification of NFAg31A. Figure S2. Sequence alignment among NFAg31A and its homologous ?-glucosidases. Figure S3. Thermostability of NFEg31A at 39 ℃, 45 ℃ and 50 ℃ [file 12934_2023_2124_MOESM1_ESM.docx]

**Microbial Cell Factories**

Supplementary file

**Insight into broad substrate specificity and synergistic contribution of a fungal α-glucosidase in Chinese Nong-flavor daqu**

**Zhuolin Yi^a^, Lanchai Chen^b^, Yanling Jin^a^, Yi Shen^c^, Nian Liu^d^, Yang Fang^a^, Yao Xiao^e^, Xi Wang^c^, Kui Peng^d^, Kaize He^a^, Hai Zhao^a*^**

^a^ CAS Key Laboratory of Environmental and Applied Microbiology, Environmental Microbiology Key Laboratory of Sichuan Province, Chengdu Institute of Biology, Chinese Academy of Sciences, Chengdu 610041, China

^b^ School of Food and Bioengineering, Xihua University, Chengdu 610039, Sichuan, China

^c^ Sichuan Langjiu Co., Ltd, Gulin 646523, China

^d^ Sichuan Food and Fermentation Industry Research & Design Institute, Chengdu 611130, China

^e^ Analytical and Testing Center, Sichuan University of Science and Engineering, Zigong 643000, China

***Corresponding author:**

Hai Zhao, CAS Key Laboratory of Environmental and Applied Microbiology, Environmental Microbiology Key Laboratory of Sichuan Province，Chengdu Institute of Biology, Chinese Academy of Sciences, No. 9 Section 4, Renmin Nan Road, Chengdu, 610041, Sichuan, P.R. China. Phone: +86-28-82890725; Fax: +86-28-82890733; E-mail: [zhaohai@cib.ac.cn](mailto:zhaohai@cib.ac.cn).

Supplementary Table S1. Accession numbers of all those selected enzymes for Phylogenetic tree analysis in four subgroups of GH31 family

| Subgroup of GH31 | Enzymes | Activities | Accession numbers |
| --- | --- | --- | --- |
| 1 | Sugar beet AG | α-glucosidase | O04931 |
|  | Spinach AG | α-glucosidase | O04893 |
|  | Barley AG | α-glucosidase | Q43763 |
|  | Pine AX | α-xylosidases | Q8VWV9 |
|  | Potato AG | α-glucosidase | Q9LEC9 |
|  | *A.niger* AG | α-glucosidase | P56526 |
|  | *S. pombe* AG | α-glucosidase | Q9C0Y4 |
|  | *A.implicatum* AG | α-glucosidase | BAD08418 |
|  | *M.javanicus* AG | α-glucosidase | Q92442 |
|  | Human NtMGAM | maltase-glucoamylase | 2QMJ |
|  | Human NtSI | sucrase-isomaltase | 3LPO |
|  | Human CtSI | sucrase-isomaltase | NP_001032 |
|  | Human CtMGAM | maltase-glucoamylase | 3TOP |
|  | *T.pyriformis* AG | α-glucosidase | O00906 |
|  | *C.thermophilum* AG II | α-glucosidase II | 5DKX |
|  | *S.cerevisiae* AG II | α-glucosidase II | CAA85192 |
|  | *P.purpureum* AG II | α-glucosidase II | KAA8491331 |
|  | *A.thaliana* AG II | α-glucosidase II | NP_201189 |
|  | Mouse AG II | α-glucosidase II | Q8BHN3 |
|  | Human AG II | α-glucosidase II | Q14697 |
|  | *B.mori* AG II | α-glucosidase II | BAM78681 |
|  | *S.frugiperda* AG II | α-glucosidase II | BAM78680 |
|  | *B.thetaiotaomicron* AG-A | α-glucosidase | 5DJW |
|  | *B. thermoamyloliquefaciens* AG | α-glucosidase | Q9F234 |
|  | *T.acidophilum* AG | α-glucosidase | CAC11443 |
|  | *P.arsenaticum* AG | α-glucosidase | ABP51590 |
|  | *P.torridus* AG | α-glucosidase | AAT42677 |
|  | *S.solfataricus* AG | α-glucosidase | 2G3M |
|  | *S.acidocaldarius* AG | α-glucosidase | AAY80507 |
|  | *L.johnsonii* AG | α-glucosidase | ACO57638 |
| 2 | *G. lemaneiformis* GL | α-glucan lyases | Q9STC1 |
|  | *M. costata* GL | α-glucan lyases | Q9UVZ2 |
|  | *M. vulgaris* GL | α-glucan lyases | Q9UVZ1 |
| 3 | *E.faecalis* AaGal | alpha-N-acetylgalactosaminidase | 6M77 |
|  | *S. solfataricus* AX | α-xylosidases | Q9P999 |
|  | *E.coli* Sul | sulfoquinovosidase | NP_418314 |
|  | *C.japonicus* ATG | α-transglucosylase | ACE84782 |
| 4 | *L. lactis* AG | α-glucosidase | CAL98407 |
|  | *P.saltans* AGal | α-galactosidase | BAR72452 |
|  | *B.thetaiotaomicron* AG-B | α-glucosidase | 5F7C |
|  | *E.coli* AX | α-xylosidases | P31434 |
|  | *L.pentosus* AX | α-xylosidases | P96793 |

Supplementary Table S2. The top 30 highest expression of genes related to carbohydrate-active enzymes at the highest temperature stage (N3) of NF daqu

| GeneID | RPKM | Class | Class_description |
| --- | --- | --- | --- |
| 31847 | 655.5 | GH31 | alpha-glucosidase, alpha-xylosidase, alpha-glucan lyase |
| 17772 | 568.6 | GT30 | α-3-deoxy-D-manno-octulosonic-acid (KDO) transferase |
| 11929 | 549.0 | GH0 | glycoside hydrolases not yet assigned to a family. |
| 8756 | 465.3 | GH3 | beta-glucosidase, 1,4-beta-xylosidase,alpha-L-arabinofuranosidase |
| 17445 | 381.2 | CBM18/GH16 | endo-1,3-beta-glucanase,xyloglucanase,endo-beta-1,3-galactanase |
| 44876 | 341.2 | GH11 | xylanase |
| 38358 | 330.3 | GH3 | beta-glucosidase, xylan 1,4-beta-xylosidase, alpha-L-arabinofuranosidase |
| 24092 | 327.5 | CE0 | carbohydrate esterases not yet assigned to a family |
| 12023 | 316.1 | GH16 | endo-1,3-beta-glucanase, xyloglucanase, endo-beta-1,3-galactanase |
| 3131 | 305.4 | GH61（AA9） | copper-dependent polysaccharide monooxygenases |
| 23023 | 304.2 | GT2 | cellulose synthase,chitin synthase |
| 17558  （NFAmy13A） | 293.5 | GH13 | alpha-amylase |
| 22702 | 290.3 | GH16 | endo-1,3-beta-glucanase,xyloglucanase,endo-beta-1,3-galactanase |
| 26838 | 288.9 | GT2 | cellulose synthase, chitin synthase |
| 21154 | 286.9 | CBM18/GH16 | endo-1,3-beta-glucanase,xyloglucanase,endo-beta-1,3-galactanase |
| 30117 | 264.9 | GT41 | UDP-GlcNAc: peptide beta;-N-acetylglucosaminyltransferase |
| 11999 | 257.4 | CBM43/GH72 | beta-1,3-glucanosyltransglycosylase |
| 27126 | 242.5 | GH27 | alpha-galactosidase,alpha-N-acetylgalactosaminidase,beta-L-arabinopyranosidase |
| 34442 | 220.5 | GT2 | cellulose synthase,chitin synthase |
| 25682 | 199.3 | GH17 | endo-1,3-beta-glucosidase, licheninase |
| 7437  （NFEg16A） | 191.2 | GH16 | endo-1,3(4)-beta-glucanase |
| 32957 | 187.1 | GH15/GT5 | glucoamylase |
| 36180 | 184.8 | GH3 | alpha-arabinosidase |
| 22572 | 137.9 | GH16 | endo-1,3(4)-beta;-glucanase |
| **15963**  **（NFAg31A）** | 127.1 | GH31 | alpha-glucosidase |
| 22903 | 125.3 | GH17 | endo-1,3(4)-beta-glucanase |
| 39884 | 119.1 | GH17 | endo-1,3(4)-beta-glucanase |
| 22243 | 117.6 | GH15/CBM20 | glucoamylase |
| 40736 | 116.1 | GH76 | alpha-1,6-mannanase |
| 15654 | 72.3 | CE4 | chitooligosaccharide deacetylase |

Supplementary Table S3. The relative high expression of alpha-glucosidase (GH31) genes at the high temperature stage (N3), and their relative expressions at the beginning stage (N1), the increasing temperature stage (N2) and mature stage (N4) of NF daqu.

| Gene ID | N1-RPKM | N2-RPKM | N3-RPKM | N4-RPKM |
| --- | --- | --- | --- | --- |
| 31847 | 0 | 0 | 655.5 | 18.9 |
| **15963**  **（NFAg31A）** | 0 | 7.8 | 127.1 | 13.1 |
| 22494 | 0 | 0 | 46.5 | 12.4 |
| 45442 | 0 | 0 | 32.8 | 4.8 |
| 47817 | 0 | 1.2 | 21.0 | 4.1 |
| 4132 | 0 | 0.5 | 16.1 | 0.5 |
| 7364 | 0 | 0.6 | 15.5 | 2.5 |
| 519 | 0 | 0 | 14.8 | 1.4 |
| 38401 | 0 | 0 | 5.1 | 1.4 |
| 56876 | 0 | 0 | 2.4 | 1.3 |
| 53489 | 0 | 0 | 0.5 | 0 |
| Total | 0 | 10.1 | 937.3 | 60.4 |

Supplementary Table S4. Effect of metal ions and chemicals on the activity of NFAg31A.

| Additives | Relative activity (%) (mean ± standard deviation) | |
| --- | --- | --- |
|  | 1 mM | 10 mM |
| Control | 100±3 | 100±2 |
| CaCl2 | 96.5±1.1 | 53.9±2.4 |
| NiCl2 | 97.3±1.4 | 44.0±2.1 |
| AlCl3 | 76.0±4.3 | 16.8±1.3 |
| BaCl2 | 108±2 | 72.9±7.8 |
| MgCl2 | 112±3 | 73.8±4.1 |
| KCl | 91.5±0.8 | 79.2±5.7 |
| CoCl2 | 105±5 | 79.1±4.0 |
| FeCl3 | 1.34±0.60 | 0 |
| MnCl2 | 50.7±3.9 | 33.2±2.0 |
| ZnSO4 | 19.8±0.6 | 12.1±2.2 |
| CuSO4 | 1.72±0.12 | 0 |
| HgCl2 | 7.11±1.34 | 3.32±0.45 |
| EDTA·Na2 | 64.0±2.8 | 40.7±1.4 |
| NH4Cl | 73.7±1.1 | 77.9±3.4 |
| SDS | 13.6±0.9 | 3.82±0.19 |
| LiCl | 96.7±2.1 | 97.0±1.6 |
| NaCl | 103±1 | 95.2±1.2 |

Note: Each experiment was performed in triplicate.

Supplementary Table S5. The synergistic hydrolysis of soluble starch by different molar ratios and combinations of NFAg31A and two alpha-amylases (NFAmy13A and NFAmy13B). Different molar ratio of NFAmy13A:NFAmy13B:NFAg31A, namely 1:1:1, 3:7:10, and 1:19:20, and their different combinations were used to hydrolyze soluble starch here. The reducing products were analyzed with *p*HBAH method. Enzyme combinations were applied as following, A: NFAmy13A; B: NFAmy13B; C: NFAg31A; AB: NFAmy13A+NFAmy13B; AC: NFAmy13A+NFAg31A; BC: NFAmy13B+NFAg31A; ABC: NFAmy13A+NFAmy13B+NFAg31A.

| Reducing end (mM) | Molar ratios (NFAmy13B : NFAmy13A : NFEg31A) | | |
| --- | --- | --- | --- |
|  | 1:1:1 (0.5μM:0.5μM:0.5μM) | 3:7:10 (0.3μM:0.7μM:1.0μM) | 1:19:20 (0.05μM:0.95μM:1.0μM) |
| **A** | 0.61±0.22 | 0.57±0.09 | 0.72±0.05 |
| B | 2.70±0.09 | 1.40±0.05 | 0.76±0.04 |
| C | 0.07±0.01 | 0.16±0.04 | 0.14±0.01 |
| AB | 2.84±0.06 | 1.60±0.04 | 1.39±0.22 |
| AC | 0.22±0.03 | 0.40±0.07 | 0.53±0.07 |
| BC | 2.42±0.01 | 1.46±0.09 | 0.78±0.06 |
| ABC | 2.82±0.02 | 1.94±0.07 | 1.24±0.06 |

Supplementary Table S6. The end products of synergistically hydrolyzing soluble starch by NFAg31A and two alpha-amylases (NFAmy13A and NFAmy13B) at molar ratio of 3:7:10. The end products were analyzed with HPAEC-PAD.

| Enzyme combination | End-products (μM) | | | | | Total malto-oligosaccharides of G1−M5 (μM) |
| --- | --- | --- | --- | --- | --- | --- |
|  | G1 | M2 | M3 | M4 | M5 |  |
| **A** | 23±4 | 322±29 | 211±20 | 98±6 | 26±4 | 679±62 |
| B | 0±0 | 80±1 | 268±5 | 126±2 | 0±0 | 474±8 |
| C | 21±1 | 0±0 | 2±0 | 8±4 | 0±0 | 32±6 |
| AB | 52±2 | 357±3 | 231±2 | 102±3 | 35±7 | 776±17 |
| AC | 42±1 | 88±2 | 533±3 | 52±5 | 40±4 | 274±15 |
| BC | 45±2 | 69±3 | 260±7 | 130±2 | 2±3 | 506±17 |
| ABC | 127±1 | 331±12 | 248±9 | 125±5 | 46±6 | 876±33 |


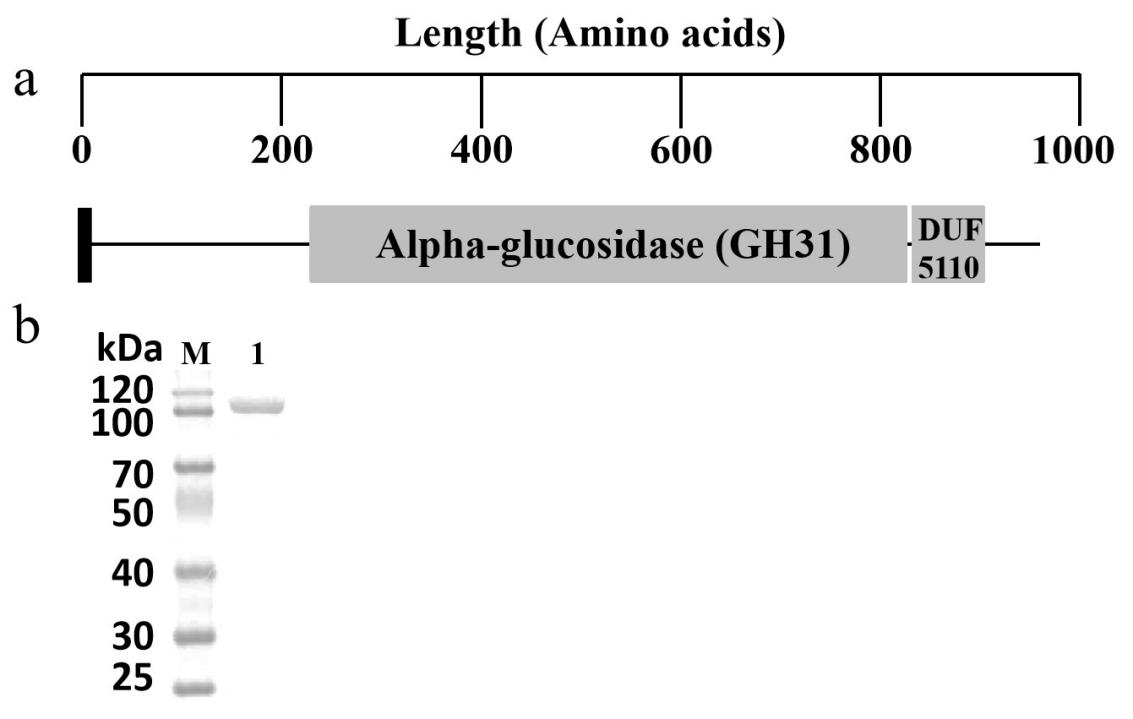


Supplementary Figure S1. Schematic representation and purification of NFAg31A. A: Schematic representation of NFAg31A; B: SDS-PAGE analysis of the purified recombinant NFAg31A. The signal peptide is shown in the filled rectangle. GH13: family 13 glycoside hydrolase domain. GUF5100: domain of unknown function 5100. Lane M: molecular mass markers; Lane 1: the purified recombinant NFAg31A.


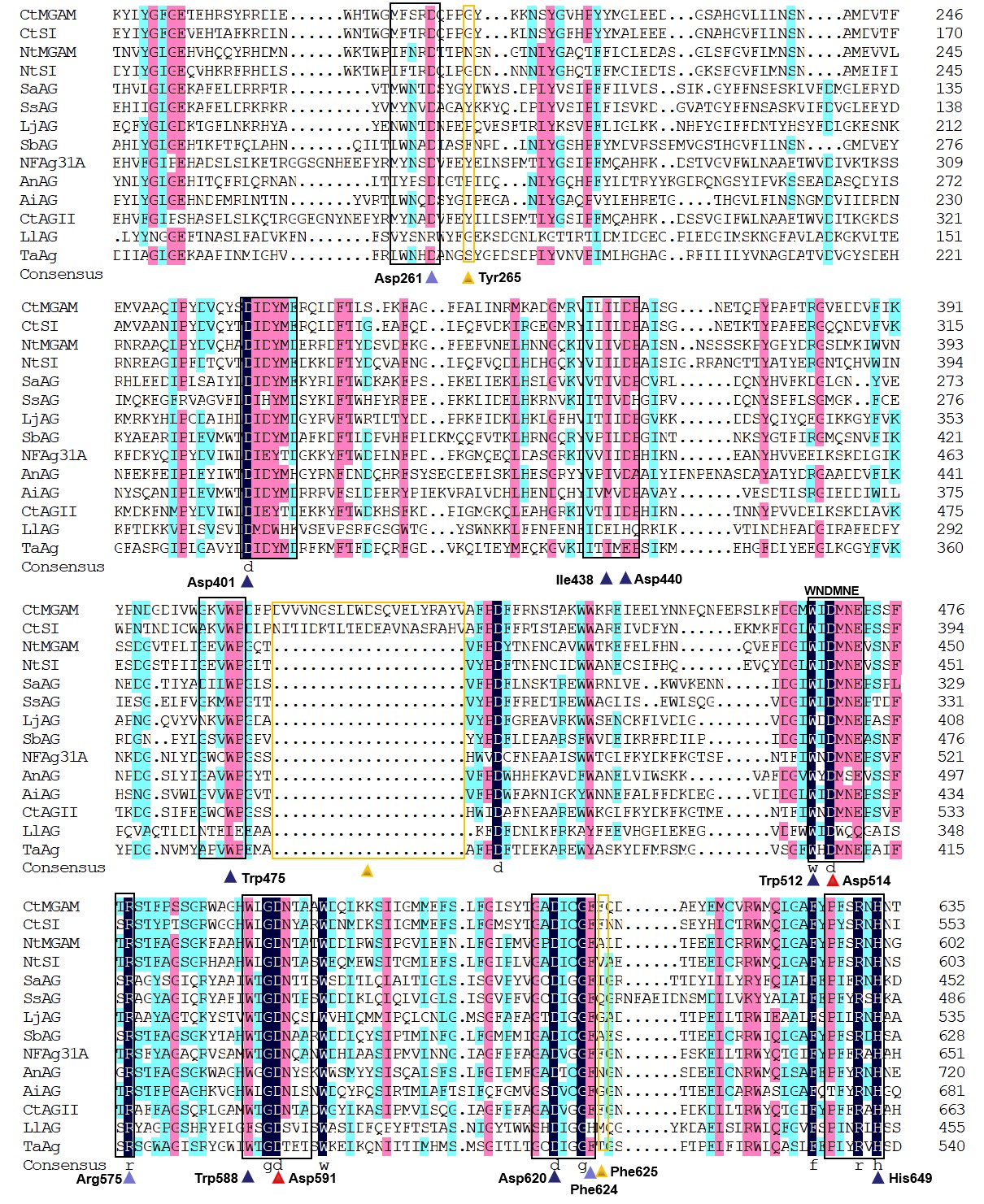


Supplementary Figure S2. Sequence alignment among NFAg31A and its homologous **α**-glucosidases. Red triangles indicate catalytic sites, blue and light blue indicates residues which interact with Glc-1 and Glc+1, respectively.The conserved motif of ‘WNDMNE’ in NFAg31A was also marked with solid rectangle. Meanwhile, those sites and sequence which might contribute to the different substrate specificity is highlighted with yellow triangle.


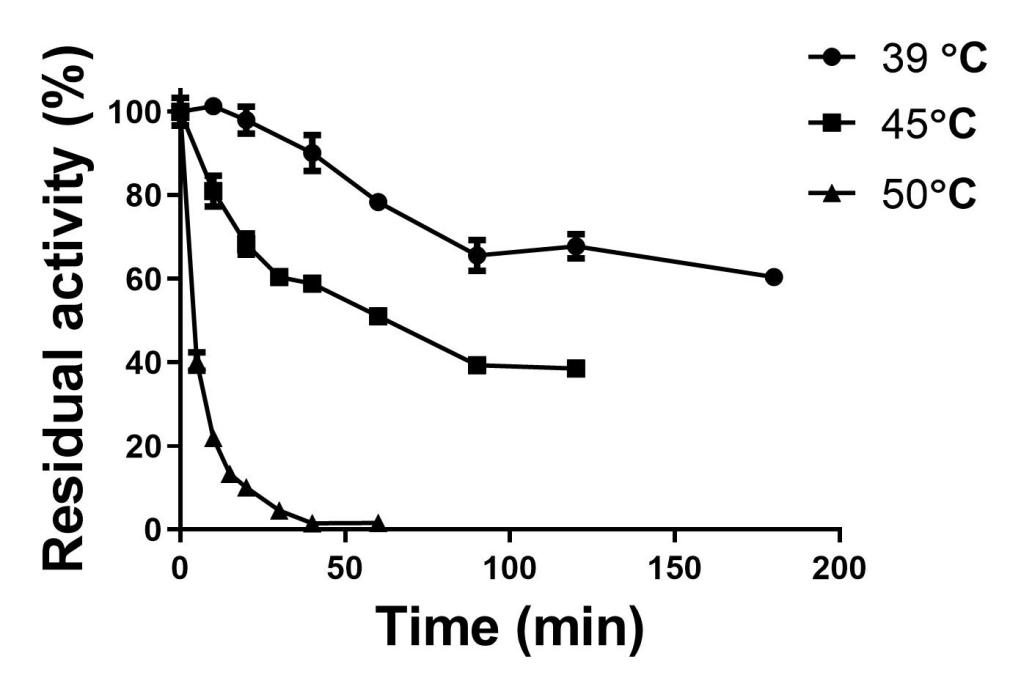


Supplementary Figure S3. Thermostability of NFEg31A at 39℃, 45℃ or 50℃. Experiments were performed by incubating NFEg31A at 39℃, 45℃ or 50℃ for different times, and their residual activities were similarly detected under standard condition. Each experiment was performed in triplicate.
